# Molecular subclassification of gastrointestinal cancers based on cancer stem cell traits

**DOI:** 10.1186/s40164-021-00246-x

**Published:** 2021-11-13

**Authors:** Mei-Mei Li, Jun Yuan, Xin-Yuan Guan, Ning-Fang Ma, Ming Liu

**Affiliations:** 1 Affiliated Cancer Hospital and Institute of Guangzhou Medical University, Guangzhou, 510095 China; 2 Guangzhou Municipal and Guangdong Provincial Key Laboratory of Protein Modification and Degradation, School of Basic Medical Science, Guangzhou Medical University, Xinzao, Panyu District, Guangzhou, 511436 China; 3 State Key Laboratory of Oncology in Southern China, Collaborative Innovation Center for Cancer Medicine, Sun Yat-Sen University Cancer Center, Guangzhou, 510060 China; 4 Department of Clinical Oncology, State Key Laboratory of Liver Research, University of Hong Kong, Hong Kong, China

**Keywords:** Gastrointestinal cancer, Heterogeneity, Cancer stem cell, Cancer subtype, Precision oncology

## Abstract

Human gastrointestinal malignancies are highly heterogeneous cancers. Clinically, heterogeneity largely contributes to tumor progression and resistance to therapy. Heterogeneity within gastrointestinal cancers is defined by molecular subtypes in genomic and transcriptomic analyses. Cancer stem cells (CSCs) have been demonstrated to be a major source of tumor heterogeneity; therefore, assessing tumor heterogeneity by CSC trait-guided classification of gastrointestinal cancers is essential for the development of effective therapies. CSCs share critical features with embryonic stem cells (ESCs). Molecular investigations have revealed that embryonic genes and developmental signaling pathways regulating the properties of ESCs or cell lineage differentiation are abnormally active and might be oncofetal drivers in certain tumor subtypes. Currently, multiple strategies allow comprehensive identification of tumor subtype-specific oncofetal signatures and evaluation of subtype-specific therapies. In this review, we summarize current knowledge concerning the molecular classification of gastrointestinal malignancies based on CSC features and elucidate their clinical relevance. We also outline strategies for molecular subtype identification and subtype-based therapies. Finally, we explore how clinical implementation of tumor classification by CSC subtype might facilitate the development of more effective personalized therapies for gastrointestinal cancers.

## Introduction

Worldwide, gastrointestinal cancers rank among the most frequent malignancies and are responsible for more than half of all cancer deaths. Common cancers of the gastrointestinal tract include liver, colorectal, pancreatic, gastric and esophageal malignancies. Current therapeutic treatments are ineffective, as most patients develop metastasis, resistance to radiation/chemotherapy, and recurrence. Thus, new strategies to improve treatment effects for patients with gastrointestinal cancers are urgently needed [1, 2].

Genomic and transcriptomic analyses reveal that human gastrointestinal malignancies are highly heterogeneous cancers. Clinically, heterogeneity largely contributes to tumor progression, metastasis, resistance to therapy, and relapse. Bulk tumors contain diverse tumor cell subpopulations with distinct molecular signatures that display differential levels of sensitivity to treatments. Under therapeutic stress, the expansion of intrinsic subpopulations or the evolution of drug-tolerant cells can lead to resistance to treatment. In clinical pathology, these kinds of subpopulations, drug-tolerant cells or poorly differentiated tumors usually exhibit stem-like traits and lead to adverse clinical events. Cancer stem cells (CSCs) have been demonstrated to be a major source of tumor heterogeneity. CSCs are a very heterogeneous subpopulation of “stem-like” cancer cells described as “tumor-initiating cells” or “sphere-forming cells” and share critical features with embryonic stem cells (ESCs), including multilineage differentiation, self-renewal and maintenance of the pluripotency state [3–5]. Specifically, the existence of molecular subtypes indicates the presence of molecular heterogeneity. Elucidation of gastrointestinal cancer classifications integrating CSC properties is critical, as such classifications may allow us to not only better understand the mechanisms of carcinogenesis from a CSC perspective but also improve diagnosis and prognostication and facilitate the development of precision medicine through identification of subtypes that may respond to specific targeted therapies. In the present review, we describe recent advances in the molecular classifications of five common gastrointestinal cancer types from the CSC perspective and elucidate their therapeutic and clinical relevance, thereby providing an overview of molecular subclassification by cancer stem cell traits for translation into clinical implementation and treatment selection.

## Gastrointestinal tumor heterogeneity and therapeutic resistance

### Tumor heterogeneity, therapeutic resistance, and cancer stem cell properties

Tumor heterogeneity consists of intertumor (tumor by tumor) and intratumor (within each tumor) heterogeneity. Tumor heterogeneity can arise from cells of origin. For example, PDAC (90% of all cases) and pancreatic neuroendocrine neoplasm (PanNEN, 3–5% of all cases) are two major histological subtypes of pancreatic cancer. PanNEN is further divided into well-differentiated pancreatic neuroendocrine carcinoma and poorly differentiated pancreatic neuroendocrine carcinoma (PanNEC). The heterogeneity among PDAC, PanNEC and PanNET can be manifested by different driver genes. The critical driver gene mutations found in PDAC include those in KRAS, CDKN2A, TP53, and SMAD4. PanNEC harbors mutations in KRAS, TP53 and RB1, while the core driver gene mutations in PanNET include alterations in MEN1, DAXX/ATRX, and mTOR pathway genes (PTEN, TSC2 and PIK3CA), which completely differ from those in PDAC and PanNET. Furthermore, the origins of PDAC, PanNEN and PanNEC are complicated. Precursor cells of intralobular ducts or acinar cells with exocrine secretion can give rise to PDAC. PanNETs may originate from the α-cell lineage, β-cell lineage or islet cell precursors. PanNEC cells of origin may arise from undifferentiated progenitor cells and harbor stem cell-like properties [6]. Accumulating evidence suggests that CSCs originate from nonmalignant stem or progenitor cells [7, 8]. CSC heterogeneity has been demonstrated to be a major source of intratumor heterogeneity within each tumor population and contributes to inducing chemoresistance and subsequent tumor relapse [9–14]. Diverse subpopulations of CSCs show distinct functions, developmental statuses or gene expression profiles [15, 16]. Cellular surface markers are a useful tool to isolate and identify CSC populations. Most of the markers are derived from hematopoietic and embryonic stem cells. Some markers have been proposed as preferential stemness markers, such as Nanog, Sox2, Oct4 and c-Myc. Some markers have been described to define CSC populations in different cancer types (Table 1); for instance, the combination of CD24 and CD44 markers delineates a common CSC population for colorectal cancer, liver cancer, pancreatic cancer, and others. Interestingly, this population also characterizes the mesenchymal-like CSC population in breast cancer [17]. In addition, the expression of most CSC markers varies between tumor types and even between patients under the same subtype. For instance, CD24 showed significantly lower expression in oral squamous cell carcinoma, while CD24 had higher expression in pancreatic intraepithelial neoplasia [18]. However, marker functionality and CSC identification are still under debate because of the lack of consistency. A possibility that heterogeneity remains in purified populations remains, and the combination of multiple markers may promote optimal CSC enrichment. Indeed, EpCAM, CD166 and CD44 were more robust as markers of colorectal carcinoma (CRC) CSCs than CD133 alone [19].

**Table 1:** Representative markers of gastrointestinal CSCs

Liver cancer	Colorectal cancer	Pancreatic cancer	Gastric cancer	Esophageal cancer
(1) CD133^+^ [91]	(1) ALDH^high^ [101]	(1) CD133^+^/ CXCR4^+^ [114]	(1) CD44^+^ [119]	(1) CD44^+^ [130]
(2) CD13^+^ [92]	(2) Lgr5^+^ [102]	(2) ALDH1A1^+^ [115]	(2) CD44v8-10^+^ [120]	(2) Integrin α7^+^ [131]
(3) EpCAM^+^ [93]	(3) ABCG2^+^/OCT4^+^ [103]	(3) pAKT^+^/SOX9^+^ [116]	(3) Snail^+^ [121]	(3) ALDH1^+^ [132]
(4) SOX9 [94]	(4) CD44v2^+^ [104]	(4) FAM83A^+^ [117]	(4) Lgr5^+^ [122]	(4) ALDH1A1^+^ [133]
(5) Lin28B^+^ [95]	(5) CD44v6^+^ [105]	(5) CD133^+^/CD44^+^/CD24^+^/ESA^+^ [118]	(5) Frizzled7^+^ [123]	(5) B7H4^+^ [134]
(6) β-catenin^+^/GEP [96]	(6) CD133^+^ [106]	(6) CD44^+^/CD24^+^/EpCAM^+^ [118]	(6) CD47^+^ [124]	(6) Gli1^+^ [135]
(7) CD133^+^/CD49f^+^ [97]	(7) CD166^+^ [107]		(7) CD133^+^ [125]	(7) Musashi1^+^ [136]
(8) CD90^+^/CD45^−^, CD44^+^/CD90^+^ [98]	(8) EpCAM^−^ [108]		(8) ALDH^+^ [126]	(8) Epiregulin^+^ [137]
(9) CD44^+^/CD133^+^ [99]	(9) E-cadherin^−^ [109]		(9) CD44^+^/CD24^+^ [127]	(9) Numb^+^ [138]
(10) SALL4^+^/EpCAM^+^ [100]	(10) CD133^+^/CD44^+^/ALDH1^+^ [110]		(10) CD44^+^/CD133^+^ [128]	(10) WASH^+^ [139]
	(11) EpCAM^+^/CD44^+^/CD166^+^ [19]		(11) CD44^+^/Snail1^+^/Vimentin^+^/E-cadherin^+^ [129]	(11) CD47^+^/CD133^+^ [140]
	(12) CD44^+^/CD24^+^ [111]			(12) CD133^+^/CXCR4^+^ [141]
	(13) CD133^+^/CXCR4^+^ [112] (14) CD133^+^/CD24^+^ [113]			

CSCs display many features of ESCs because they tend to retain activation of one or several vital and highly conserved signaling pathways involved in the differentiation and pluripotency of stem cell phenotypes. CSCs can cause and sustain tumor growth, similar to ESCs, which develop into blastocysts and provide sustenance for fetal growth. They can both generate tumor cells from various stem cells and normal somatic cells. In addition, they have similar putative transcription factors (e.g., Nanog, Sox2, Oct4, Klf4, and c-Myc) and surface markers (e.g., CD133, CD90, CD24, and CD44). Furthermore, they are enriched in developmental signaling pathways regulating the features of embryonic cells or normal organogenesis or cell lineage differentiation, which may drive the initiation and progression of poorly differentiated malignancies. Five major signaling pathways have been identified as bestowing embryonic stemness traits upon tumor cells. These pathways included the Hedgehog, Hippo, Notch, TGF-β and Wnt/β-catenin pathways. All these pathways play important roles in conferring the ability of CSCs to turn into identical daughter cells by self-renewal, thereby maintaining immortality and differentiating into various types of cells. Moreover, these pathways are involved in gastrointestinal cancer initiation, migration and resistance. As CSCs are highly heterogeneous, the expression of stemness pathways varies at different time points and in different gastrointestinal tumor types. Interestingly, activation of CSC pathways can also be identified in tumor cell expressing distinct CSC markers. For example, overexpression of Notch1 and Notch2 has been correlated with increased expression of CD44 and EpCAM in pancreatic cancer (PDAC) [20]. Wnt signaling has been shown to be activated to maintain the self-renewal and tumorigenicity of CD44+ gastric CSCs [21]. In HCC, Notch and Jagged have been shown to be highly expressed in CD133+ hepatocellular carcinoma (HCC) CSCs [22]. To date, an increasing number of biomarkers indicating activation of CSC pathways are being discovered continuously.

**Fig. 1:**
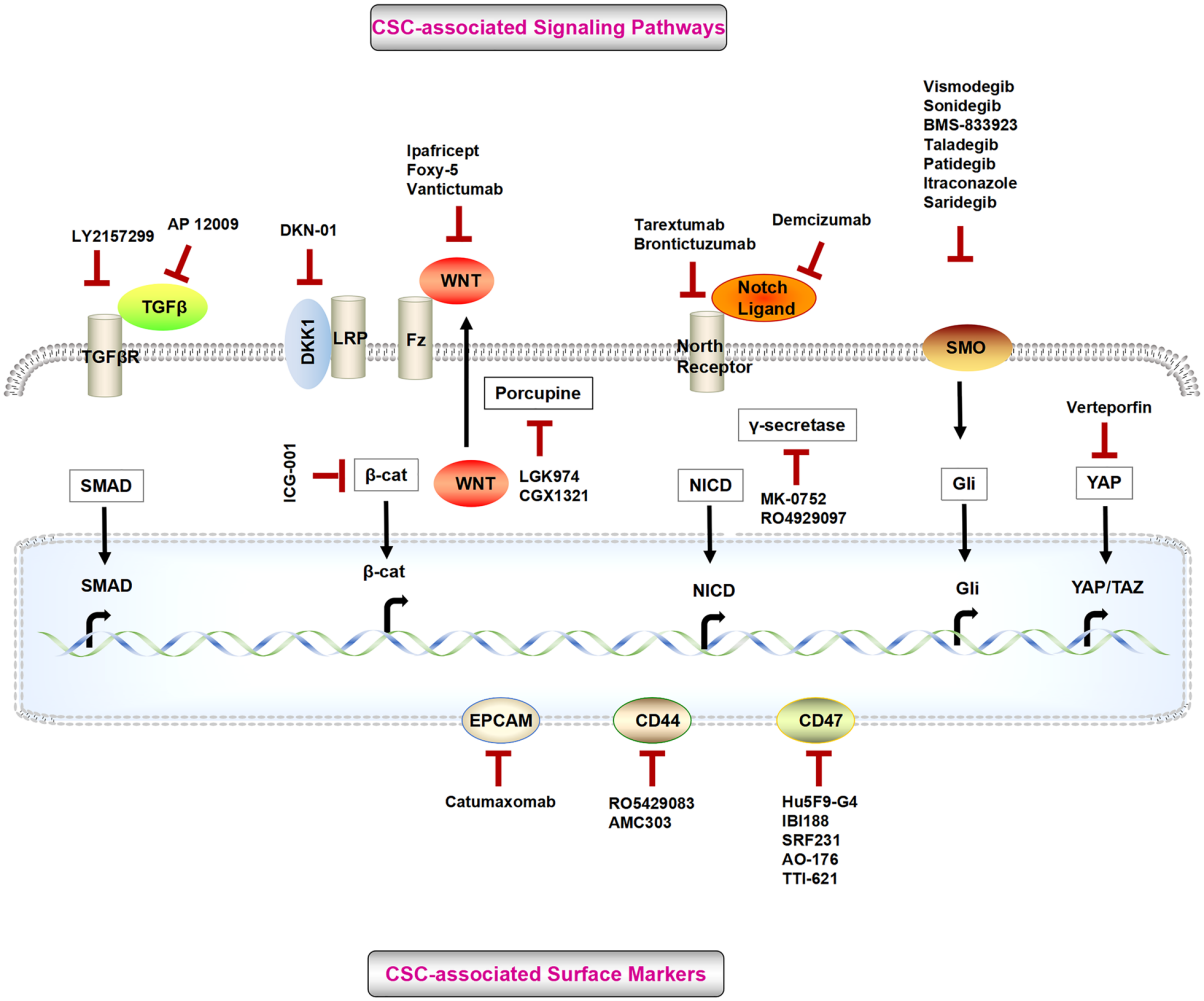
CSCs-targeted therapies. Selected anti-CSC drugs targeting developmental pathways and CSC-associated surface markers, which are involved in clinical trials. TGFβ: transforming growth factor; TGFβR: transforming growth factor receptor; LRP: low-densitylipoprotein-related protein; Fz: frizzled; β-cat: β-catenin; NICD: Notch intracellular domain; SMO: smoothened

CSC biomarkers and signaling pathways are critical factors distinguishing molecular classifications with stem-like traits. The expression levels plus the activation degrees of CSC biomarkers and signaling pathways differ in different subtypes, which has led to investigations into potential new avenues of targeted therapy. CSC-targeted therapies are currently in development, and many are already in clinical trials (Fig. 1; Table 2). To achieve better clinical outcomes, combination-based therapies should be implemented in CSC-targeting strategies. Moreover, CSC-directed therapy should be applied preferably early when CSC populations are still small and resistance pathways have not yet been induced. CSC-directed therapy can also be applied in various stages of the patient treatment journey.

**Table 2:** CSC-targeting agents

Signaling pathways	Cancer types	Therapeutic agents	Targets	Phase	Combination drugs	Reference
TGF-β signaling						
Antisense oligonucleotides	Pancreatic neoplasms, Colorectal Neoplasms	AP 12009 (Trabedersen)	TGF-β2	Phase 1		NCT00844064
TβR kinase inhibitors/small-molecule inhibitors	(1) Hepatocellular carcinoma (2) Advanced or metastatic unresectable pancreatic cancer (3) Rectal adenocarcinoma (4) Advanced hepatocellular carcinoma (5) Advanced hepatocellular carcinoma (6) Metastatic cancer and advanced or metastatic unresectable pancreatic cancer (7) Metastatic pancreatic cancer (8) Advanced refractory solid tumors; Hepatocellular carcinoma (9) Metastatic pancreatic cancer	LY2157299 (Galunisertib)	TβRI	(1) Phase 1 (2) Phase 1 (3) Phase 2 (4) Phase 2 (5) Phase 2 (6) Phase 1b/2 (7) Phase 1 (8) Phase 1b/2 (9) Phase 1	(1) Sorafenib (2) Galunisertib (3) Capecitabine, Fluorouracil (4) Sorafenib (5) Sorafenib, Ramucirumab (6) Gemcitabine (7) Durvalumab (8) Nivolumab (9) Durvalumab	(1) NCT02240433 (2) NCT02154646 (3) NCT02688712 (4) NCT02178358 (5) NCT01246986 (6) NCT01373164 (7) NCT02734160 (8) NCT02423343 (9) NCT02734160
Wnt signaling						
β-catenin inhibitors	(1) Advanced pancreatic cancer; Metastatic pancreatic cancer; Pancreatic adenocarcinoma (2) Hepatitis C virus-infected cirrhosis (3) Colorectal adenocarcinoma; Stage IVA colorectal cancer; Stage IVB colorectal cancer	ICG-001(PRI-724)	CBP/β-Catenin	(1) Phase 1 (2) Phase 1 (3) Phase 2	(1) Gemcitabine (3) Bevacizumab, Leucovorin, Oxaliplatin, Fluorouracil	(1) NCT01764477 (2) NCT02195440 (3) NCT02413853
Wnt antibodies	(1) Hepatocellular carcinoma (2) Pancreatic cancer; Stage IV pancreatic cancer	Ipafricept (OMP-54F28)	Fzd8-Fc fusion protein	(1) Phase 1 (2) Phase 1	(1) Sorafenib (2) Nab-Paclitaxel Gemcitabine	(1) NCT02069145 (2) NCT02050178
	(1) Colorectal cancer (2) Metastatic colon cancer	Foxy-5	WNT5a receptor	(1) Phase 1 (2) Phase 1		(1) NCT02020291 (2) NCT02655952
Wnt antibodies	Pancreatic cancer	Vantictumab (OMP-18R5)	Frizzled receptor	Phase 1	Nab-Paclitaxel and Gemcitabine	NCT02005315
DKK1 antibodies	(1) Hepatocellular carcinoma (2) Esophageal neoplasms; Adenocarcinoma of the gastroesophageal junction; gastroesophageal cancer; Gastric adenocarcinoma (3) Carcinoma of intrahepatic and extra-hepatic biliary system; Bile duct cancer; Cholangiocarcinoma	DKN-01	DKK1	(1) Phase 1/2 (2) Phase 1 (3) Phase 1	(1) Sorafenib (2) Paclitaxel or pembrolizumab (3) Gemcitabine and cisplatin	(1) NCT03645980 (2) NCT02013154 (3) NCT02375880
Porcupine inhibitors	(1) Metastatic colorectal cancer (2) Pancreatic cancer; Esophageal squamous cell cancer	LGK974 (WNT974)	Porcupine	(1) Phase 1 (2) Phase 1	(1) LGX818, Cetuximab (2) PDR001	(1) NCT02278133 (2) NCT01351103
	Colorectal adenocarcinoma; Gastric adenocarcinoma; Pancreatic adenocarcinoma; Bile duct carcinoma; Hepatocellular carcinoma; Esophageal carcinoma; Gastrointestinal cancer	CGX1321	Porcupine	Phase 1	Pembrolizumab	NCT03507998
Notch signaling						
DLL-4 antibody	(1) Metastatic pancreatic ductal adenocarcinoma (2) Locally advanced or metastatic pancreatic cancer (3) Colorectal cancer	Demcizumab (OMP-21M18)	DLL4	(1) Phase 2 (2) Phase 1 (3) Phase 1	(1) Gemcitabine, Abraxane® (2) Gemcitabine, Abraxane®	(1) NCT02289898 (2) NCT01189929 (3) NCT01189942
Notch receptor antibody	Untreated stage IV pancreatic cancer	Tarextumab (OMP-59R5)	Notch2, Notch3	Phase 1/2	Nab-Paclitaxel, Gemcitabine	NCT01647828
	Metastatic colorectal cancer	Brontictuzumab (OMP-52M51)	Notch1	Phase 1	Trifluridine or tipiracil	NCT03031691
γ-secretase inhibitor	Pancreatic cancer	MK-0752	γ-secretase	Phase 1	Gemcitabine hydrochloride	NCT01098344
γ-secretase inhibitor	(1) Metastatic pancreas cancer (2) Metastatic colorectal cancer (3) Metastatic colorectal cancer (4) Metastatic colorectal cancer	RO4929097 (R4733)	γ-secretase	(1) Phase 2 (2) Phase 2 (3) Phase 1 (4) Phase 2	(2) FOLFOX regimen, Bevacizumab, Oxaliplatin, leucovorin calcium, fluorouracil (3) Cetuximab	(1) NCT01232829 (2) NCT01270438 (3) NCT01198535 (4) NCT01116687
Hedgehog signaling						
SMO inhibitor	(1) Metastatic pancreatic adenocarcinoma (2) Metastatic colorectal cancer (3) Pancreatic adenocarcinoma (4) Metastatic pancreatic cancer or solid tumors (5) Pancreatic ductal adenocarcinoma (6) Metastatic colorectal cancer (7) Recurrent or metastatic pancreatic cancer	Vismodegib (GDC-0449)	SMO	(1) Phase 2 (2) Phase 2 (3) Phase 1 (4) Phase 1 (5) Phase II (6) Phase II (7) Phase II	(1) Gemcitabine, nab-paclitaxel (2) Bevacizumab, Modified FOLFOX, FOLFIRI (3) Gemcitabine (4) Erlotinib, gemcitabine (6) Vismodegib, FOLFOX, FOLFIRI, Bevacizumab (7) Gemcitabine hydrochloride	(1) NCT01088815 (2) NCT00636610 (3) NCT01713218 (4) NCT00878163 (5) NCT01096732 (6) NCT00959647 (7) NCT01064622
	Metastatic gastric, gastroesophageal, esophageal adenocarcinomas	BMS-833923 (XL139)		Phase 1	Cisplatin, capecitabine	NCT00909402
	Esophageal cancer	Taladegib (LY2940680)		Phase 1/2	Paclitaxel, carboplatin, radiation	NCT02530437
	(1) Metastatic pancreatic cancer (2) Advanced pancreatic adenocarcinoma	Patidegib (IPI-926)		(1) Phase 1 (2) Phase 1	(1) Gemcitabine (2) FOLFIRINOX	(1) NCT01130142 (2) NCT01383538
SMO inhibitor	(1) Esophageal cancer (2) Esophageal Cancer (3) Locally Advanced Squamous Esophageal Cancer	Itraconazole	SMO	(1) Phase 1 (2) Phase 2 (3) Phase 2		(1) NCT02749513 (2) NCT04018872 (3) NCT04481100
	(1) Pancreatic cancer (2) Pancreatic cancer	Saridegib (IPI-926)		(1) Phase 1 (2) Phase 1/2	(1) 5-fluorouracil, Leucovorin, Irinotecan, Oxaliplatin (2) Gemcitabine	(1) NCT01383538 (2) NCT01130142
	(1) Resectable pancreatic adenocarcinoma (2) Advanced or metastatic HCC (3) Esophageal cancer (4) Advanced pancreatic cancer (5) Pancreatic cancer (6) Pancreatic cancer (7) Pancreatic cancer (8) Resectable pancreatic cancer	Sonidegib (LDE225)		(1) Phase 1/2 (2) Phase 1 (3) Phase 1 (4) Phase 1 (5) Phase 1/2 (6) Phase 1 (7) Phase 1 (8) Phase 1	(1) Gemcitabine, nab-paclitaxel (3) Everolimus (4) Fluorouracil, leucovorin, oxaliplatin, irinotecan (5) Gemcitabine, nab-paclitaxel (6) Gemcitabine (7) Fluorouracil; Leucovorin; Oxaliplatin; Irinotecan	(1) NCT01431794 (2) NCT02151864 (3) NCT02138929 (4) NCT01485744 (5) NCT02358161 (6) NCT01487785 (7) NCT01485744 (8) NCT01694589
Hippo signaling						
YAP inhibitor	Pancreatic cancer non-resectable	Verteporfin	YAP	Phase 2	Photodynamic therapy	NCT03033225
CSC surface markers						
Anti-CD44 antibody	Malignant solid tumor	RO5429083	CD44	Phase 1		NCT01358903
CD44v6 inhibitor	Malignant solid tumor	AMC303	CD44v6	Phase 1		NCT03009214
Anti-CD47 antibody	(1) Colorectal neoplasms/Solid tumors (2) Advanced malignancies (3) Advanced solid cancers (4) Solid tumor	(1) Hu5F9-G4 (2) IBI188 (3) SRF231 (4) AO-176	CD47	(1) Phase 1 (2) Phase 1 (3) Phase 1 (4) Phase 1/2	(1) Cetuximab	(1) NCT02953782 (2) NCT03763149 (3) NCT03512340 (4) NCT03834948
Recombinant fusion protein binding CD47	Solid tumor	TTI-621	CD47	Phase 1	Rituximab or Nivolumab	NCT02663518
Anti-EpCAM antibody	(1) Gastric cancer, Gastric adenocarcinoma (2) Gastric Adenocarcinoma With Peritoneal Carcinomatosis, Siewert Type II/III Adenocarcinoma of Esophagogastric Junction With Peritoneal Carcinomatosis	Catumaxomab	EpCAM	(1) Phase 2 (2) Phase 2		(1) NCT00464893 (2) NCT01504256

### Tumor plasticity

Cancer cell plasticity has been proposed as one of the important mechanisms contributing to intratumor heterogeneity. Plasticity enables cancer cells to shift between a nontransformed differentiated state and a tumorigenically transformed undifferentiated or CSC state in response to microenvironmental stimuli (e.g., oncogenic stresses, senescence, and inflammation). Plasticity usually includes stem cell multilineage interconversion, dedifferentiation and transdifferentiation [23, 24]. CSCs may arise from their normal stem cells, progenitors and/or differentiated somatic cells. CSCs have the potential to differentiate into cancer cells, dedifferentiate into original lineage cells, and/or transdifferentiate into other lineage cells [25, 26]. Aberrantly activated plasticity drives malignant transformation and confers tumors to accommodate the constraints of tumor growth and therapy resistance. In our previous study, we found that CHD1L (chromodomain-helicase-DNA-binding protein 1-like gene) is a potential clinical developmental lineage oncogene in HCC. CHD1L expression is active in the embryonic stage but decreases progressively during terminal differentiation. However, CHD1L expression is abnormally amplified in HCC. This dynamic expression pattern is accompanied by elevated liver ancestral precursor markers and reduced hepatic lineage differentiation markers. Further suppression of CHD1L may hinder poorly differentiated HCC and sensitize patients to chemotherapeutic drugs [27]. Our recently published study [28] found another oncofetal driver, Claudin6 (CLDN6), which has a dynamic expression pattern similar to that of CHD1L, as a potential therapeutic target correlated with HCC lineage plasticity. A high CLDN6 abundance led to a phenotypic shift of the HCC cellular subtype from a hepatic lineage to a biliary lineage, which confers sorafenib resistance. A de novo anti-CLDN6 monoclonal antibody (CLDN6-DM1) specific for CLDN6+ cells was developed. The preclinical data demonstrated the potent therapeutic efficacy of CLDN6-DM1 as a single agent or in combination with sorafenib in HCC management.

Epithelial-to mesenchymal transition (EMT) is one of the most important processes of plasticity. The EMT program is dynamic and fundamental to embryonic development [29]. Accumulating evidence has revealed the connection between CSCs and EMT. Although whether EMT is necessary for CSCs is still under debate, EMT undoubtedly plays an important role in CSCs. First, at earlier stages of progression, depending on the microenvironmental cues, the intermediate mesenchymal states are reversible, and EMT in tumor cells may be transient, which leads to a greater plastic CSC phenotype, poorer patient survival and greater resistance to chemotherapy. For example, six different populations of EpCAM-cells identified by the CSC markers CD61, CD106 and CD51 exhibited this intermediate EMT state and more efficiently formed metastases [30]. Second, elevation of EMT master transcription factors not only enforces metastatic potential but also exacerbates the tumor-initiating capacity [9, 31, 32]. Indeed, most gastrointestinal cancer subtypes with stem cell features display a strong association with the EMT phenotype.

## Identification of molecular subtypes with CSC properties in gastrointestinal malignancies

Gastrointestinal malignancies are highly heterogeneous within tumors and have been defined by identifying so-called molecular subtypes. Transcriptomic, genomic, and/or epigenomic profiling of many tumors offers the basis for molecular classification. These distinct molecular subtypes reflect different biological backgrounds, including immunity, metabolism, and stemness. Specifically, CSCs have been demonstrated to be a major source of intratumor heterogeneity. Integrative analyses of molecular subclassification from the CSC perspective may be encouraged with the aim of determining consensus molecular classification in patient prognostication and selection for therapies (Table 3).

**Table 3:** Gastrointestinal cancer subclassifications with CSC traits

Cancer types	Reference	Classifications	Signatures & Pathways (+: Activation, −: Suppression)	Prognosis	Inhibitors (+: Sensitive, −: Resistance)
Liver cancer	Zhu et al. [30]	Notch-high	Notch and TGF-β signaling, KRT19, DCLK1, SOX9 (+)	higher tumor stage, worse survival outcome	
	Liu et al. [31]	ES^+^, LP^+^	ES^+^: pluripotency and stem cell self-renewal signaling pathways (+), Gli, Notch and Wnt pathways (+); LP^+^: TGF-β, Notch and Wnt pathways (+)		ES^+^: HLM6474 (+); LP^+^: SIS3 (+)
	Lee et al. [32]	HB, HC	HB: KRT7, KRT19, VIM, AP-1 complex (+)	HB: tumor-invasive, worse survival, poorer prognosis	
	Yamashita et al. [33]	HpSC-HCC (EpCAM^+^AFP^+^), MH-HCC (EpCAM^−^AFP^−^)	HpSC-HCC: KRT19, c-Myc, Wnt/β-catenin (+); MH-HCC: HepPar1 (+)	HpSC-HCC: tumor-invasive, poor prognosis MH-HCC: good prognosis	HpSC-HCC: β-catenin inhibitor (+); EpCAM^+^ HCC cells: GSK-3β inhibitor BIO, 5-FU (−); EpCAM^−^ HCC cells: 5-FU (+)
	Hoshida et al. [34]	S1, S2, S3	S1: Wnt, TGF-β, EMT (+); S2: Myc, AKT, EpCAM, AFP (+); S3: hepatocyte differentiation (+)	S1: earlier recurrence, tumor-invasive, poor survival; S2: poor survival; S3: good survival	
	Boyault et al. [35]	G1, G2, G3, G4, G5, G6	G1: AXINI mutations, fetal liver expressing genes, AFP (+); G2: AKT (+); G3: cell cycle genes (+); G5: Wnt (+); G6: Wnt, LEF1 (+), CDH1 (−)	G4-G6: better survival *VS* G1-G3	
Colorectal cancer	Marisa et al. [36]	C1, C2, C3, C4, C5, C6	C1: EMT (−); C2: Wnt (−); C3: EMT (−); C4: EMT (+); C5: Wnt (+); C6: EMT (+)	C4 plus C6: worse prognosis *VS* all other subtypes	
Colorectal cancer	Sadanandam et al. [37]	Enterocyte, goblet-like, inflammatory, transit-amplifying (CS-TA, CR-TA), stem-like	Goblet-like and enterocyte: differentiation markers (+), stem cell and Wnt markers (−); Transit-amplifying: stem and progenitor markers (+), Wnt-target genes (−); Stem-like: Wnt, stem cell, myoepithelial and mesenchymal markers (+), differentiation markers (−)	Goblet-like and transit-amplifying: good prognosis; Enterocyte and inflammatory: intermediate DFS; Stem-like tumors: shortest DFS	Goblet-like: cetuximab (+); Inflammatory: FOLFIRI (+); Stem-like: cetuximab, FOLFIRI (+); CS-TA: cetuximab (+); CR-TA: cMET inhibitors (+), cetuximab (−)
	De Sousa et al. [38]	CCS1, CCS2, CCS3	CCS1: Wnt (+); CCS3: EMT, matrix remodeling and TGF-β (+)	CCS3: poor prognosis	CCS3: cetuximab (−)
	Budinska et al. [39]	Surface crypt-like, lower crypt-like, CIMP-H-like, mesenchymal, mixed	Surface crypt-like: EMT (−),Wnt (−), β-catenin (−); Lower crypt-like: EMT (−), Wnt (+); CIMP-H-like: β-catenin (−); Mesenchymal: EphB2, EMT (+), Wnt (−), β-catenin (−); Mixed: EphB2, EMT, Wnt (+)	Surface crypt-like and lower crypt-like: better prognosis *VS* mesenchymal; CIMP-H-like: poor OS and SAR; Mesenchymal: recurrence risk, poor OS; Mixed: poorer SAR *VS* lower crypt-like	
	Roepman et al. [40]	Type A, Type B, Type C	Type A: EMT (−); Type B: EMT (−); Type C: EMT (+)	Type A: good prognosis; Type B: poor prognosis; Type C: poor prognosis	Type A: 5-FU (+); Type B: 5-FU (+); Type C: 5-FU (-)
	Guinney et al. [41]	CMS1, CMS2, CMS3, CMS4	CMS1 and CMS3: RTK and MAPK pathways (+); CMS2: HNF4A, Myc and Wnt (+); CMS4: EMT, TGF-β, angiogenesis, matrix remodeling pathways (+)	CMS1: worse SAR; CMS4: worse RFS and OS	CMS1: HSP90 inhibitors (+); CMS2: HSP90 inhibitors, EGFR inhibitors, HER2 inhibitors (+); CMS4: combination treatment of 5-FU and luminespib (+)
Pancreatic cancer	Collisson et al. [43]	Classical, QM-PDA, exocrine-like	Classical: GATA6 (+); QM-PDA: mesenchyme associated genes (+)	Classical: good prognosis; Exocrine-like: intermediate prognosis; QM-PDA: worst prognosis	Classical: erlotinib (+), docetaxel (−); QM-PDA: oxaliplatine, 5-FU, BET inhibitor (+); Exocrine-like: SN-38 (−)
	Moffitt et al. [44]	Normal stromal, activated stroma, basal-like, classical	Activated stroma: SPARC, WNT2, WNT5A, MMP9, MMP11 (+); Classical: GATA6 (+);	Activated stroma: worse survival *VS* normal stroma; Basal-like: worse survival *VS* classical	Normal stroma: Hedgehog pathway inhibitor (+); Basal-like: oxaliplatine, 5-FU, BET inhibitor (+); Classical: docetaxel, SN-38 (−)
	Bailey et al. [45, 81]	Pancreatic progenitor (PP), squamous, immunogenic and aberrantly differentiated endocrine exocrine (ADEX)	Squamous: pancreatic endodermal cell-fate determination genes, Hedgehog/Wnt pathway (−); TGF-β and Myc pathway (+); PP: developmental transcription factors (+), Notch pathway (+) ADEX: pancreatic developmental and differentiational genes (+), Notch pathway (+)	PP: good survival outcomes; Immunogenic and ADEX: intermediate survival outcomes; Squamous: worst survival outcomes	Squamous: oxaliplatine, 5-FU, BET inhibitor, GSK3β inhibitor (+); Squamous and PP subtypes: docetaxel (−); PP: SN-38 (−)
	Biederstädt et al. [80]	Squamous/basal- like	SUMO pathway and Myc (+)	Worse prognosis	SUMOylation inhibitor (+)
	Mueller et al. [46]	Cluster 1, Cluster 2, Cluster 3, Cluster 4, Cluster 5	Cluster 1: squamous differentiation; Cluster 2: epithelial differentiation; Cluster 3: embryonic development, EMT, MAPK pathway; Cluster 5: embryonic development		
Pancreatic cancer	Puleo et al. [47]	Pure classical, immune classical, pure basal-like, stroma activated, desmoplastic	Stroma activated and pure basal-like: MET, Hedgehog pathway (+)	Pure classical and immune classical: good prognosis; Stroma activated and desmoplastic: poor prognosis; Pure basal-like: worst prognosis	
	Sivakumar et al. [48]	Notch, repressed Hedgehog/Wnt, cell cycle		Notch: best prognosis; Repressed Hedgehog/Wnt: worst prognosis	
	Seino et al. [49]	W^+^, W^−^, WRi	W^+^: exogenous Wnt (−), R-spondin (+); W^−^: exogenous Wnt (+), R-spondin (+); WRi: Wnt signaling (−)	W^+^: poor survival and metastatic progression	
Gastric cancer	Lei et al. [53]	Proliferative, metabolic, mesenchymal	Proliferative: E2F, Myc, RAS (+); Mesenchymal: EMT, CSC pathway (+)	No significant differences among the 3 subtypes	Metabolic: 5-FU (+); Mesenchymal: PI3K-AKT-mTOR inhibitors (+)
	Cristescu et al. [54]	MSI, MSS/EMT, MSS/p53^+^ and MSS/p53^−^	MSS/EMT: EMT (+)	MSS/EMT: worst prognosis, recurrence, stage III/IV, earlier age; MSI: stage I/II, best prognosis	
	Cheul Oh et al. [55]	EP, MP	EP: Wnt (+), EMT (−); MP: EMT, IGF pathway, Hedgehog pathway (+)	EP: better survival; MP: poor survival	EP: adjuvant chemotherapy (+); MP: adjuvant chemotherapy (−), IGF1/IGF1R pathway inhibitor (+)
	Cheong et al. [56]	Epithelial, immune, stem-like	Epithelial: CDX1 (+); Stem-like: SFRP4 (+)	Low-risk (immune-high), intermediate-risk (immune-low and stem-like-low), or high risk (immune-low and stem-like-high)	No-benefit (immune-high or immune-low and epithelial-low) or chemotherapy-benefit (immune-low and epithelial-high)
Oesophageal cancer	Walker et al. [57]	ESCC1, ESCC2, ESCC3	ESCC1: SOX2, TP63 (+); ESCC2: ZNF750 and Notch1 mutation, CDK6 amplification (+), KDM6A and KDM2D (−), PIK3R1 and PTEN (−) ESCC3: mutations associated with RTK/RAS/PI3K pathway (+)		
	Wang et al. [58]	Subtype I, Subtype II	Subtype II: epithelium development genes (+)	No significant differences between the 2 subtypes	
	Jammula et al. [59]	Subtype 1, Subtype 2, Subtype 3, Subtype 4	Subtype 1: DNA repair and cell cycle driver genes (+); Subtype 2: differentiational and developmental transcription factors (+); Subtype 4: high level of copy number alterations (+)		Subtype 1: CDK4/6 inhibitors, docetaxel (+); Subtype 2: CDK4/6 inhibitors (+); Subtype 3: CDK4/6 inhibitors (+); Subtype 4: CDK4/6 inhibitors (+), CDK2 inhibitors (+);

ADEX: aberrantly differentiated endocrine-exocrine; CCS: colon cancer subtype; CIMP: CpG island methylator phenotype; CMS: consensus molecular subtype; EMT: epithelial-tomesenchymal transition; EP: epithelial phenotype; EpCAM: epithelial cell adhesion molecule; ES: embryonic stem cell; ESCC: oesophageal squamous cell carcinomas; FU: 5-fluorouracil; HB: hepatoblasts; HC: hepatocytes; LP: liver progenitor cell; MP: mesenchymal phenotype; MSI: microsatellite instability; MSS: microsatellite-stable; OS: overall survival; PH: premature hepatocytes; PP: pancreatic progenitor; QM-PDA: quasi-mesenchymal-pancreatic ductal adenocarcinoma; RFS: relapse-free survival; RTK: receptor tyrosine kinase; SAR: survival after relapse; VS: versus

### Classification with CSC properties in liver cancer

Zhu et al. [33] used a 14-gene Notch score to stratify HCC into Notch-high HCC and Notch-low HCC subtypes. The Notch-high HCC subtype was associated with less differentiated tumors and poor survival, characterized by increased expression of progenitor/cholangiocyte markers (DCLK1 and KRT19), and highly enriched in genes related to developmental signaling and the fetal liver. In contrast, Notch-inactive HCC is a subtype of well-differentiated neoplasms with a better prognosis. In our recently published study [34], human ESCs were differentiated into human hepatocytes, and the whole differentiation process was defined by four stages: embryonic stem cells (ESs), endoderm (EN), liver progenitor cells (LPs), and premature hepatocytes (PHs). We classified liver cancer into two major subtypes based on oncofetal gene expression patterns. We defined the genes from the ES and EN groups as the embryonic-like subtype (ES+ subtype) and genes from the LP and PH groups as the liver progenitor-like subtype (LP+ subtype). Interestingly, the ES+ subtype was mainly associated with genes in the pluripotency and stem cell self-renewal signaling pathway and the Gli signaling pathway, while the LP+ subtype was mainly associated with the TGF-β signaling pathway. Moreover, genes in the Notch and Wnt signaling pathways span all four stages. Lee et al. [35] uncovered two subgroups in their study: hepatocytes (HCs) and hepatoblasts (HBs). The specific HB subtype may arise from adult hepatic progenitor cells and features elevated expression of KRT7 and KRT19. Based on the oncofetal gene expression profiling, Yamashita et al. [36] distinguished two HCC subtypes: HpSC-HCC (referred to as EpCAM+ AFP+) and MH-HCC (referred to as EpCAM- AFP-). KRT19 and Wnt/β-catenin signaling are enriched in EpCAM+ AFP+ HCC cells. The EpCAM+ subgroup of HCC displayed a similar expression pattern to the LP+ tumors in our study. In Hoshida et al.’s study [37], HCC patients were classified into S1, S2 and S3 subgroups based on the extent of tumor differentiation. The Wnt pathway was activated in S1 tumors by a mechanism of TGF-β signature activation. Class S2 was a progenitor cell group featuring Myc and AKT activation and EpCAM and AFP enrichment. S3 tumors were notable for differentiated hepatocyte function. Most of the patients’ tumors with oncofetal properties in our study were consistent with the poorly differentiated S1 and S2 subgroups. Accordingly, the nononcofetal class of HCC matched the well-differentiated S3 subtype. Boyault et al.’s study [38] divided patients into G1 through G6 subgroups according to clinical and genetic characteristics. G1 and G2 tumors were characterized by AKT activation and fetal liver properties, G3 tumors were typified by activation of cell cycle genes, heterogeneous G4 tumors were associated with rare TCF1 mutations, and G5 and G6 tumors were strongly related to Wnt pathway activation.

### Classification of CSC properties in colorectal cancer

Similar to other gastrointestinal cancers, considerable effort has been dedicated to colorectal cancer stemness-based subtyping. Marisa et al. [39] revealed six subtypes: C1 (21%) is characterized by suppression of pathways associated with EMT, C2 (19%) is characterized by suppression of the Wnt pathway, C3 (13%) is characterized by suppression of EMT, C4 (10%) often shows upregulation of EMT and genes related to stem cell-like signatures, C5 (27%) exhibits overexpression of Wnt pathway genes, and C6 (10%) shows upregulation of the EMT pathway. Studies performed by Sadanandam et al. [40] identified five subtypes and proposed that the five subtypes were associated with distinct cell subtypes found in normal colonic crypts. These subtypes are referred to as enterocyte, goblet-like, inflammatory, transit-amplifying, and stem-like subtypes. The transit-amplifying subtype is a heterogeneous subtype highly enriched for stem cell-relevant genes and the Wnt pathway and can be further divided into two groups based on the differential cetuximab response (CS-TA and CR-TA). Another stem-like subset is characterized by overexpression of Wnt signaling target genes and the presence of mesenchymal and myoepithelial stem-cell features, with downregulation of differentiation markers, whereas the goblet-like and enterocyte subsets are enriched in well-differentiated genes with few stem cell characteristics and low Wnt marker expression. In De Sousa et al. [41], they revealed three colon cancer subtypes: CCS1, CCS2 and CCS3. CCS1 (49%) refers to tumors with high activity of the Wnt signaling cascade, while CCS3 (27%) corresponds to heterogeneous and poorly differentiated tumors with upregulation of EMT, matrix remodeling and the TGF-β pathway. Unlike traditional molecular classification according to gene expression profiling, Budinska et al. [42] applied meta-gene profiles to identify five major subsets: surface crypt-like, lower crypt-like, CIMP-H-like, mesenchymal and mixed. Surface crypt-like and lower crypt-like subtypes are well differentiated with low expression of the EMT/stoma gene module when the mesenchymal subtype and the mixed subtypes are enriched for high expression of the EMT/stroma gene module. In addition, the lower crypt-like and mixed subsets highly expressed Wnt signaling target signatures along with higher β-catenin nuclear immunoreactivity. In contrast, surface crypt-like and mesenchymal subgroups showed low expression of these signatures along with lower β-catenin nuclear immunoreactivity. Moreover, the CIMP-H-like subtype exhibited almost no β-catenin nuclear immunoreactivity and low expression of gut development genes. Another classification based on whole-genome analysis of CRC patients in stages I-IV was discovered by Roepman et al. [43], who unveiled three molecular subtypes: Type A, Type B and Type C. Type A (22%) corresponds to a DNA mismatch repair (MMR)-deficient epithelial subtype, Type B (62%) represents an epithelial proliferative subtype, and Type C (16%) is characterized by the expression of EMT-related molecules. Intriguingly, these three subtypes overlapped with the three subtypes distinguished by De Sousa et al.

The above CRC subtyping systems considered three to six molecular subtypes with different characteristics that might lack compatibility and lead to some confusion. To standardize the different molecular subtypes, a large-scale study of 4000 CRC samples mainly in stages II-III was performed [44] to identify four distinct molecular classifications that correctly classified 78% of the samples: CMS1 (14%, MSI immune), CMS2 (37%, canonical), CMS3 (13% metabolic) and CMS4 (23% mesenchymal). CMS2 is characterized by epithelial differentiation and strong activation of the Wnt and Myc signaling pathways. CMS4 is characterized by EMT upregulation, activation of TGF-β signaling, enhanced matrix remodeling, complement-mediated inflammation and angiogenesis. In addition, NOTCH3 is a putative target for advanced CMS4 CRC patients [45]. CMS1-4 may reasonably be similar to any of the molecular subtypes mentioned above. CMS1 may fit the CCS2 class from De Sousa and the inflammatory class from Sadanandam. The class CMS2 consensus may be related to the CCS1 subtype from De Sousa and to the enterocyte and/or transit-amplifying subtypes from Sadanandam. The CMS4 subset can be associated with CCS3 tumors from De Sousa and with the stem-like module defined by Sadanandam.

### Classification of CSC properties in pancreatic cancer

Collisson et al.’s study [46] described three subtypes: classical, quasi-mesenchymal (QM-PDA) and exocrine-like. The classical subtype is enriched in GATA6, while the QM-PDA subtype has comparatively low GATA6 expression. GATA6 is essential for pancreatic development and differentiation. Moffitt et al. [47] extended the work from Collisson et al. by defining two subtypes for the tumor tissue (classical and basal-like) while adding stromal classifications (normal and activated). The classical subtype overlaps with Collison’s classical subtype and is characterized by elevated GATA6 expression. In addition, “activated” stroma is characterized by genes relevant to tumor promotion, such as the secreted protein SPARC, MMP family members MMP9 and MMP11, and WNT family members WNT2 and WNT5A. More recently, Bailey et al. [48] defined four molecular subtypes of PDAC: pancreatic progenitor (PP), squamous, immunogenic and aberrantly differentiated endocrine-exocrine (ADEX). The squamous subset entails downregulation of genes that control pancreatic endodermal cell fate determination, repression of Hedgehog/Wnt signaling, and TGF-β signaling and MYC pathway activation. The PP subtype is characterized by developmental transcription factors and enrichment of Notch signaling. The ADEX class is enriched with genes that are important in lineage specification and later stages of pancreatic development and differentiation. Intriguingly, a spectrum of differentiation that resembles embryonic lineages from early progenitors to fully differentiated cells exists in these subtypes. The Collison and Bailey classifications overlapped fairly well, with the exception of the immunogenic subtype. The Collison classical subtype is similar to the PP subtype, the QM-PDA subtype is similar to the squamous subtype, and the exocrine-like subtype is similar to the ADEX subtype. Following Bailey et al.’s study, Mueller et al. [49] defined five distinct clusters based on evolutionary trajectories and KRAS gene dosage. Cluster 1 resembles the squamous subtype. Clusters 2 and 5 are associated with epithelial cell differentiation and embryonic development. Cluster 3 is enriched for undifferentiated tumors and characterized by EMT and Ras downstream signaling. Cluster 4 is enriched for undifferentiated tumors and corresponds to the immunogenic subtype. Another subtyping study from Puleo et al. [50] further distinguished five subtypes: pure classical, immune classical, pure basal-like, stroma activated and desmoplastic. The pure classical subtype is well differentiated (low-grade G1) and similar to the classical and PP subtypes. The pure basal-like subtype is poorly differentiated (high-grade G3) and associated with metastatic spread. The other subtypes correspond to an intermediate differentiation grade. The immune classical and desmoplastic subtypes fit Moffitt’s ‘normal stroma’ subtype, while the pure basal-like and stroma activated subtypes fit Moffitt’s ‘activated stroma’ subtype. Furthermore, the MET and Hedgehog signaling pathways are both activated in the stroma and pure basal-like subtypes.

Researchers have also classified PDAC based on CSC-related signal transduction pathways. In Sivakumar et al.’s study [51], three main biological processes generated by the transcriptional signatures of oncogenic KRAS-specific master regulators were identified: Notch, repressed Hedgehog/Wnt, and the cell cycle. All three subtypes represent three different transcriptional programs during PDAC development and are linked to the Bailey subtypes. Suppression of Hedgehog/Wnt signaling is involved in the squamous subtype, Notch signaling is enriched in the ADEX and PP subtypes, and the cell cycle process is overrepresented by samples from the immunogenic subtype. Seino et al. [52] unveiled three subtypes based on the Wnt signaling pathway from a tumor organoid library: W+ (Wnt-secreting organoids), W- (Wnt-nonsecreting organoids) and WRi (Wnt and R-spondin-independent organoids). The W+ subtype is independent of exogenous Wnt ligands but requires R-spondin, the W- subtype depends on exogenous Wnt and R-spondin ligands, and the WRi subtype is Wnt signaling-independent.

Notably, potential overlap of defined subtypes may exist. Interestingly, plasticity occurs in these subtypes; that is, one subtype can switch to another, such as squamous to ADEX conversion [53, 54]. In mouse models, tumors shifted from squamous to classical after BET inhibitor treatment [55]. Another example is GATA6-mediated subgroup switching, as GATA6 downregulation contributes to the QM-like subtype in PDAC [48]. Conversely, GATA6-high PDACs exhibit higher levels of epithelial Wnt ligands, indicating GATA6-regulated Wnt niche dependency in patients with PDACs [52].

### Classification of CSC properties in gastric cancer

Lei et al. [56] unveiled three subtypes in their study: proliferative, metabolic, and mesenchymal. The mesenchymal subtype harbors CSC-like properties with the following four features. First, this subtype is strongly associated with CSC pathway activation. Second, it shows high CD44 and low CD24 levels compared with other types, which is similar to the QM-PDA subtype of PDAC. Third, it maintains an undifferentiated state, which is an essential feature of CSCs. Finally, the hypermethylated gene sets significantly overlap with genes expressed at low levels in HCC harboring hepatic stem cell properties. In addition, the proliferative subtype shows elevated activities for several oncogenic pathways: E2F, MYC, and RAS. Cristescu et al. [57] used gene expression data to describe four patient subsets of gastric cancer: MSI, MSS/EMT, MSS/p53+ and MSS/p53− , where MSS refers to microsatellite stable tumors. The MSS/EMT module was significantly correlated with the EMT signature. Another Korean study led by Oh et al. [58] distinguished two distinct molecular subtypes: the epithelial phenotype (EP) and mesenchymal phenotype (MP). Higher recurrence rates reflecting the clinical consequences of EMT were shown for the MP subtype, as the EMT-promoting pathway (TGF-β, Hedgehog pathway) and proteins (MYH11, RICTOR and CAV11) were highly activated in the MP module. The development and progression of EP-subtype gastric tumors are mainly due to activation of the Wnt pathway through repression of the SFRP family (SFRP1, SFRP2, SFRP3, and SFRP4). A retrospective study by Cheong et al. [59] identified four classifier genes to stratify patients into three subtypes: epithelial (CDX1), immune (GZMB and WARS), and stem-like (SFRP4). SFRP4 is a modulator of Wnt signaling-associated EMT, suggesting that EMT might contribute to the clinical consequences of the stem-like subtype.

### Classification of CSC properties in esophageal cancer

Esophageal cancer includes two main histological types: esophageal adenocarcinomas (EACs) and esophageal squamous cell carcinomas (ESCCs). In contrast to studies on other gastrointestinal tract tumors, molecular classification studies of esophageal cancer are currently lacking. Walker et al. [60] unveiled three molecular subtypes: ESCC1, ESCC2, and ESCC3. ESCC1 tumors show high amplification of SOX2 and TP63. SOX2 is a pluripotent stem cell transcription factor that favors the development and maintenance of squamous epithelia. ESCC2 tumors contain more ZNF750 and NOTCH1 mutations, inactivation of the histone demethylases KDM6A and KDM2D, deactivation of the PIK3CA suppressors PIK3R1 and PTEN, and CDK6 amplification. The last group, ESCC3, contains mutations forecasting activation of the RTK/RAS/PI3K pathway. Another study performed by Wang et al. [61] revealed two distinct subtypes of ESCC. Subtype I entails a highly activated pathway involved in the immune response, while subtype II is enriched in pathways involved in ectoderm development. Epithelium development genes, including E2F4, JUN, KRT5 and KRT14, were enriched in Subtype II. PDPN and SIX1 have high expression levels in Subtype II ESCC, while SIX1 can maintain or increase PDPN-positive CSCs. Specifically, they discovered potential ESCC subset-specific diagnostic markers: EYA2 and FOXA1 for subtype I and KRT14 and LAMC2 for subtype II, which may help guide ESCC clinical treatment. Most recently, Jammula et al. [62] identified four subtypes from Barrett’s esophagus and EAC. Subtype 1 showed elevation of driver gene alterations (CCND1, CCNE1, MYC, CDK6). Subtype 2 displayed significant overexpression of sets of key master transcription factors correlated with differentiation and development, including HNF4A/G, FOXA1/2/3, GATA6 and CDX2. Subtype 3 was enriched in all pathways related to immune regulation, while subtype 4 contained a high quantity of copy number alterations. Considering the obvious parallels existing in these three classifications by Walker, Wang and Jammula, the extent of connection among the three classifications remains to be fully addressed.

## Common events of gastrointestinal subtypes

Cancer stem cell properties are included in molecular classification systems for gastrointestinal cancers. Most classifications are characterized by similar stem cell traits, poor differentiation, and poor clinical outcomes. Most gastrointestinal tumors appear to belong to subgroups with EMT traits, for example, the C4, CCS3, mesenchymal, type C and CMS4 subsets in CRC, the cluster 3 and pure basal-like subgroups in PDAC, and the MSS/EMT and MP subtypes in gastric cancer. Another consistently identified subtype is characterized by the activation of signaling pathways involved in ESC differentiation and pluripotency, such as the Wnt pathway, TGF-β pathway, Hedgehog pathway and Myc pathway. For instance, the Wnt pathway is enriched in the C5, C6, transit-amplifying, stem-like, CCS1 and CMS2 subgroups in CRC, in the ‘activated’ stroma and W+ subgroups in PDAC, and in the EP subgroup in gastric cancer. Additionally, most classifications reflect the original functions of ESCs characterized by overexpression of key developmental and differentiation factors, for example, our ES and LP subtypes in liver cancer and the PP, squamous, ADEX, Cluster 2 and Cluster 5 subgroups in PDAC.

Notably, genetic mutations also contribute to the tumor stemness phenotype. For instance, ESCC2 esophageal cancers are enriched for NOTCH1 mutations, and mutations in ESCC3 drive activation of the RTK/RAS/PI3K pathway, indicating that genomic and transcriptomic subtypes interact with each other. Integrating both genomic and transcriptomic information may help identify the related entities or entities with common origins. Furthermore, according to clinical observations of poorly differentiated gastrointestinal cancers with preserved lineage characteristics of their developmental precursor cells, such tumors may progress to acquire classifiable phenotypes, and the similarities between tumor subtypes from different organs may be defined from early embryonic development events that are reflected in the developmental signaling expression or mutational profiles of classified tumors. The inter- and intratumor heterogeneity caused by these events can be used to foster patient welfare.

## Evaluating strategies for subtype-directed therapy

### Subtyping identification strategies

The tumor heterogeneity of each subtype is mainly explored by multiomics (transcriptomics, proteomics, metabolomics, lipidomics, glycomics) in many publicly available repositories (such as TCGA, ICGC and GEO) or institutional sources. For example, Liu et al. performed unsupervised clustering to define three immune subtypes with different features from multiple HCC databases and developed a support vector machine (SVM) classifier based on multiomics signatures, and this multiomics SVM model provided potential predictors for prognosis and responses to immunotherapy in HCC [63]. Molecular subtyping typically requires tissue biopsy samples. However, subtyping strategies may be hampered by the following aspects. First, in some hardly accessible tumors, such as PDAC, omics-based subtype classifications are difficult to obtain; in this case, small classifiers can be devised to circumvent this problem by working on small amounts of tumor tissues from routine diagnostic cytology. Second, intratumor heterogeneity may lead to sampling error and possibly tumor misclassification, and developing marker panels or blood-based markers for tumor subtypes can help circumvent these problems [64]. Recently, liquid biopsy has become an appealing noninvasive clinical tool for the isolation and detection of blood-based markers. Jose et al. [65] provided an example to apply a microfluidic platform to identify CSC subtypes (CD133+CK+CD45−DAPI+EpCAM+ and CD133+CK+CD45-DAPI+EpCAM-) from patient blood samples in PDAC. Liquid biopsy can overcome the difficulties of obtaining tissue biopsies, capture spatial and/or temporal heterogeneity, and facilitate therapy response monitoring [66]. However, multiple technical issues, especially insufficient sensitivity and specificity, still need to be solved for future clinical application.

To date, markers for tumor subtypes can be measured using flow cytometry, real-time quantitative polymerase chain reaction (qPCR), and immunohistochemical or immunofluorescent staining [67]. In addition, recent achievements of single-cell techniques such as scRNA-seq (single-cell RNA sequencing) have provided extraordinary insights into intratumor heterogeneity, which has already been highlighted in cancer classification, diagnosis, and treatment [68]. scRNA-seq can be used to characterize rare but important subtypes. For example, Daniel et al. [16] revealed a novel stemness-related cell subclone (CD24+/CD44+) within EPCAM+ HCC cells, and suppression of the signature gene CTSE in CD24+/CD44+cells abrogated the self-renewal ability of HCC. Lin et al. [69] applied scRNA-seq to identify the EMT+ PDAC subtype and epithelial tumor cell (ETC) population. The reported high mesenchymal gene expression signals (i.e., QM subtype) were enriched in the EMT+ subtype, and the signature genes defining the classic, progenitor and squamous subtypes were enriched in the ETC population, whereas the signature genes defining the basal subtype were enriched in both EMT and ETC tumor cells.

### Preclinical models for subtype therapy

Various drug sensitivity studies have been performed using the most common models, such as tumor-derived cell lines and patient-derived xenografts (PDXs), which can retain the common molecular characteristics of primary tumors and generate valuable transcriptomic information for molecular subtypes and corresponding clinical and pharmacological data for association studies. Several large-scale studies have been performed on a large set of tumor-derived cell lines for biomarker discovery and drug response prediction. Stefano et al. [70] screened the most commonly used liver cancer cell lines, including 34 models, and in combination with screening 31 anticancer agents, identified markers of therapeutic response. Another promising technique for large-scale functional screening using RNAi or CRISPR/Cas9 has also been applied to study cancer subtypes. For example, Robert et al. [71] performed a large-scale RNAi screen in 398 cancer cell lines to elucidate the vulnerabilities of specific cancer subtypes. Although tumor-derived cell lines are easily manipulated and acceptable for stem cell-based subtype identification and high-throughput screening, 2D culture cannot fully reproduce the native 3D microenvironment of tumor cells. Instead, the PDX model more reliably recapitulates patient subtypes than 2D culture by retaining patient histopathological and molecular features. Researchers have successfully translated the CMS classification of CRC to preclinical PDX models for targeted treatment and distinguished patients with poor clinical consequences within the CMS groups [72–74]. However, the shortcomings of long engraftment periods and low engraftment efficiency hamper large-scale drug screening with PDX models. Alternatively, spheroids are used as important 3D preclinical models to test the effects of targeted drugs, especially to investigate the interaction between pharmacological and radiotherapeutic strategies. For example, Che et al. [75] established co-cultured pancreatic stellate cells/PDAC heterospheroids and found that this model exhibited higher resistance to gemcitabine than PDAC-only spheroids. The role of dCK in gemcitabine resistance was further studied by using this model. Another useful 3D preclinical model is organoids. Cancer-derived organoids are good in vitro models that capture tumor subtype heterogeneity, enable therapeutic screening and encompass unique subsets required for precision medicine development. Helen et al. [76] established a human gastric cancer organoid biobank that encompassing the most known molecular subtypes. Takashi et al. [52] developed a pancreatic tumor organoid library and identified three subtypes based on the stem cell niche factor associated with Wnt and R-spondin. Genetically engineered models appear to be another preclinical platform to evaluate molecular subtypes and therapeutic responses; however, they are unlikely to benefit patients whose tumors lack the target [77–80].

## Clinical relevance and subtype-driven therapies

### Liver cancer subtypes

Zhu et al. [33] used a 14-gene Notch score to sort Notch-active signatures. Notch-active HCCs were found to resemble cholangiocarcinoma (CC)-like HCC and exhibit higher tumor stages and poorer prognoses than Notch-inactive HCCs. Notch signaling is best known for its role in cell fate determination. An overwhelming number of studies have shown that Notch signaling plays promoting roles in carcinogenesis and tumor progression; therefore, patients with cancer may benefit from Notch pathway blockade. Currently, multiple Notch inhibitors against γ-secretase, Notch receptors or ligands have been developed, including γ-secretase inhibitors, siRNA and monoclonal antibodies. The combination of Notch inhibitors with other chemotherapy or radiotherapy holds considerable promise for achieving better curative effects [81]. As a detailed subclassification of stem cell-like tumors is lacking, we established new classification models to mimic the whole differentiation process from human ESCs to human hepatocytes and classified HCC patients into two subtypes based on stem-like expression patterns. E2F1 and SMAD3 are two important oncofetal drivers of liver tumors with defined gene signatures. HCC patients with the ES-like subtype were more sensitive to the E2F1 inhibitor HLM6474, while HCC patients with the LP-like subtype were more sensitive to the SMAD3 inhibitor SIS3, indicating that targeting specific oncofetal drivers may promote drug selectivity and eliminate tumorigenicity effectively [34]. Lee et al. [35] uncovered a fetal HB subtype that might arise from hepatic progenitor cells with a poor prognosis. Another stemness-based HCC classification was proposed by Yamashita and colleagues [36]. The EpCAM+ AFP+ HCC subgroup harbored progenitor features with a poor prognosis, while the EpCAM-AFP-HCC subset had adult hepatocyte features with a good prognosis. Moreover, β-catenin inhibitors were more effective in EpCAM+ HCC cells than in EpCAM- HCC cells in vitro. In addition, a GSK-3β inhibitor and 5-fluorouracil (FU) increased the EpCAM+ population in HCC cells. Based on the extent of tumor differentiation, Hoshida et al. [37] classified HCC patients into S1, S2 and S3 subgroups. Subclass S1 is linked with a higher risk of early recurrence, with more satellite lesions and vascular invasion. As TGF-β boosts Wnt activity by altering the subcellular localization of β-catenin, cotargeting TGF-β and β-catenin may be an effective strategy for the treatment of the S1 subclass of HCC. S2 tumors demonstrate Myc and AKT activation, suggesting that AKT or PI3K inhibitors might be valuable in this particular subclass. In contrast, the S3 subclass contains the majority of well-differentiated tumors, which tend to have a lower grade and better survival outcomes.

### Colorectal cancer subtypes

In Sadanandam et al.’s study [40], the goblet-like and transit-amplifying subtypes showed a good prognosis, the enterocyte and inflammatory subtypes were associated with intermediate disease-free survival (DFS), while the stem-like tumors corresponded to the shortest DFS but were shown to benefit more from FOLFIRI than others, while CS-TA and CR-TA tumors were sensitive to cetuximab and cMET inhibitor treatment, respectively. De Sousa et al. [41] compared the clinical characteristics of CCS1 and CCS3 tumors in their study and found that patients with CCS1 tumors had a good prognosis. CCS3 tumors harbored malignant potential at an early stage of adenomas and were refractory to anti-EGFR therapy. Budinska et al. [42] assessed the associations of their classifications with patient survival. Surface crypt-like and lower crypt-like subgroups showed a better prognosis. CIMP-H-like and mesenchymal subtypes were associated with poor overall survival (OS), while the former was also associated with short survival after relapse (SAR). The mixed subgroups showed a trend toward the worst OS. Molecular classification performed by Roepman et al. distinguished three subclasses [43]; Type A has the best prognosis, Type B has an intermediate prognosis but can benefit from adjuvant 5-FU chemotherapy, and Type C showed the worst survival and resistance to 5-FU-based chemotherapy. When assessing the existence of core subtype gene expression patterns among available CRC subtyping algorithms, four consensus molecular subtypes were observed to be related to clinical characteristics [44]. CMS1 patients are usually diagnosed at higher pathologic grades and show worse survival after relapse. Conversely, CMS2 patients had superior survival rates after relapse, whereas CMS4 patients had worse relapse-free and overall survival and were more likely to be in stage III and stage IV. Recently, Sveen et al. [72] translated this CMS system to preclinical models containing CRC-derived cell lines and PDX models to perform high-throughput in vitro drug screening. They found that CMS2 tumors were strongly responsive to EGFR and HER2 inhibitors and that CMS1 and CMS2 tumors were highly sensitive to HSP90 inhibitors. Furthermore, combination treatment with 5-FU and luminespib could relieve chemoresistance in CMS4 patients.

### Pancreatic cancer subtypes

In Collisson et al.’s study [46], among the three subtypes that they described, the classical subtype had a better prognosis than the other two types, while patients with QM-PDA subtype tumors had the worst prognosis. For subtype-specific drug responses, the classical subtype was more sensitive to erlotinib. Moffitt et al. [47] defined two major subtypes for the tumor (classical and basal-like) and stromal classifications (normal and activated). Patients with the activated stroma subtype had a worse median survival than those with the normal stroma subtype. Inhibition of the Hedgehog pathway could accelerate the development of PDAC and promote the delivery of chemotherapy in the normal stromal subtype. In addition, patients with the basal-like subtype had a worse medium survival than those with the classical subtype; however, the former type showed a better response to adjuvant therapy than the classical subtype. More recently, Bailey et al. [48] published four subtypes as described above. The squamous subtype is correlated with significantly worse clinical outcomes. Some patients in the PP subgroup had better survival outcomes than those in the immunogenic and ADEX subgroups. Multivariate analysis found that this classification exhibited independent prognostic value [82]. A comprehensive analysis of drug sensitivity in the above three classifications (Collisson, Moffitt and Bailey) showed that the QM-PDA, basal-like and squamous subtypes are sensitive to oxaliplatine and 5-FU. The activated stroma, QM-PDA and squamous subtypes show more resistance to gemcitabine than the ADEX subtype and Collisson's and Moffitt's classical tumor subtypes. Collisson's and Moffitt’s classical subtypes and Bailey's squamous and PP subtypes are resistant to docetaxel. PP, Collisson’s exocrine-like and Moffitt's classical subtypes are resistant to SN-38. When comparing gene expression in different subtypes, the QM-PDA, basal-like and squamous subtypes strongly expressed Myc, indicating that these subtypes may be more sensitive to BET inhibitors. EGFR signaling has been reported to enhance cancer cell stemness [83, 84]; although PDAC cells frequently present high EGFR expression, most are easily resistant to anti-EGFR treatment. Combined targeted therapy may help overcome this resistance. Moreover, Biederstädt et al. [85] detected coactivation of MYC and SUMO in the basal-like/squamous subtype of PDAC, which is known to be resistant to chemotherapies. SUMOylation inhibitor-based therapies might be a potential strategy to target this aggressive PDAC subtype. Specifically, Brunton et al. [86] found that loss of HNF4A and GATA6 could lead to a plasticity switch from the classical (progenitor) subtype to the squamous subtype and elevated expression of lycogen syn-thase kinase 3 beta (GSK3β). GSK3β inhibitors showed selective sensitivity in the squamous subtype; however, a subgroup of squamous patient-derived cell lines (PDCLs) acquired drug tolerance and had access to the WNT gene program. In addition, another developmental transcription factor, HNF1A, is a novel regulator of pancreatic cancer stem cell properties, and HNF1A + tumors (non-QM, overlap with the exocrine/ADEX subtype) benefit more from FOLFIRINOX than gemcitabine-based treatment [87]. Puleo et al. [50] redefined subtypes of PDAC into five groups. The pure classical and immune classical subclasses had similar good prognoses. The patients in the stroma-activated and desmoplastic subgroups had a severe prognosis when pure basal-like tumors had the worst outcome. The Hedgehog pathway was highly enriched in stomal activated and pure basal tumors, suggesting that Hedgehog inhibitors may help prolong survival in PDAC patients with tumors in these two subgroups. In another study [51], three subtypes generated by the transcriptional signatures of oncogenic KRAS-specific master regulators were identified: Notch, repressed Hedgehog/Wnt, and the cell cycle. Evidence of the potential clinical importance of the three groups revealed that the Hedgehog/Wnt group had the worst prognosis, while the Notch group showed the best prognosis. Seino and colleagues [52] established a library of PDAC-derived organoids and identified heterogeneous subtypes dependent on Wnt ligands. They found that epithelial Wnt molecules (Wnt3, Wnt7a, Wnt7b, and Wnt10a) could serve as a surrogate marker for Wnt-producing PDACs. Notably, WRi and W + organoids displayed higher levels of epithelial Wnt gene expression than W-organoids, and high expression of epithelial Wnt molecules was closely linked to markedly poor survival and metastatic progression.

### Gastric cancer subtypes

Lei et al. [56] developed a robust classification of primary gastric adenocarcinomas: proliferative, metabolic, and mesenchymal. Analysis of survival information showed no significant differences in survival among the three subgroups. Patients with proliferative- and mesenchymal-subtype tumors did not benefit from 5-FU treatment. In contrast, mesenchymal-subtype gastric cancer cells were preferentially sensitive to PI3K-AKT-mTOR inhibitors, possibly because this subtype of cells resembles CSCs. This finding is consistent with the observation that PI3K-AKT-mTOR inhibitors are also effective in prostate cancer and glioblastoma [88, 89]. High levels of CD44 are another distinctive feature of the mesenchymal subtype. CD44 is a well-known surface biomarker of CSCs and is aberrantly expressed in a variety of tumors in the forms of CD44s (standard isoform) or CD44v (variant isoform). A high abundance of CD44 is closely associated with a malignant phenotype and poor clinical outcomes. CD44-positive cancer cells displayed lower sensitivity to sorafenib and 5-FU. Targeting CD44 may be a promising therapeutic strategy for cancer management. CD44 antibodies and blockade of the HA-CD44 balance offer therapeutic interventions to effectively impair the properties of CSCs among various cancers [90].

Cristescu et al. [57] investigated the clinical relevance of their four molecular subtypes and found that the age at occurrence of the MSS/EMT subtype was significantly lower than that of the other subtypes. Most subjects with this subtype were diagnosed at a late stage (III/IV) and showed the worst prognosis and the highest recurrence frequency among the four subtypes. Oh et al. [58] described two subtypes: MP and EP. Clinically, the MP subtype is associated with significantly poor survival, a high recurrence rate, and resistance to standard adjuvant chemotherapy. The EP subtype is correlated with better survival and sensitivity to adjuvant chemotherapy. Importantly, MP-subtype cancer cells are significantly more sensitive to linsitinib treatment than EP-subtype cancer cells. Cheong et al. [59] uncovered three subtypes (immune, stem-like, and epithelial) for patients with resectable stage II-III gastric cancer and then developed a prognosis-based single-patient classifier to divide patients into low-risk (immune-high), intermediate-risk (immune-low and stem-like-low), or high risk (immune-low and stem-like-high) groups. They also developed a prediction-based single-patient classifier to divide patients into no-benefit (immune-high or immune-low and epithelial-low) or chemotherapy-benefit (immune-low and epithelial-high) groups. The association between the prognostic single-patient classifier groups and 5-year OS was significant. Furthermore, the association between the predictive single-patient classifier groups and adjuvant chemotherapy response in terms of OS was also notable. Collectively, the MSS/EMT, MP and stem-like subtypes have the worst prognosis in terms of clinical consequences for multiple cohorts, highlighting the significance of stemness-based subsets requiring clinical intervention.

### Esophageal cancer subtypes

Clinically, molecular classification studies of esophageal cancer are still limited. Jammula et al. [62] unveiled four subtypes relevant to therapy. Subtype 1 was sensitized to CHFR, which is a cell cycle checkpoint inhibitor. In addition, CDK4/6 inhibitors were effective across all subtypes, whereas CDK2 inhibitors were preferentially effective toward subtype 4 patients.

## Conclusions

Our understanding of gastrointestinal cancer biology has drastically improved. The main genetic changes and tumor subtypes are gradually becoming well established, and their clinical relevance is being clarified. Evident distinctions are present in the biological features and clinical properties of gastrointestinal cancers, which are probably a result of heterogeneity. Clinically, heterogeneity largely gives rise to tumor progression, metastasis, resistance to therapy, and relapse. Molecular heterogeneity arises from the existence of molecular subtypes. Due to the notable effect of CSCs on heterogeneity, CSC traits are undoubtedly tightly associated with molecular classifications. Interestingly, CSCs usually resemble embryonic stem cells, which signifies the importance of developmental signals in cancer initiation and therapeutic resistance. Therefore, integrating the molecular subtypes associated with stemness properties may offer new insights into treatment resistance.

Although molecular classifications based on CSC traits are substantially expanding our understanding of gastrointestinal malignancies, the implementation of effective precision medicine is still hindered by some problems. First, sufficient studies describing the stemness-based molecular subtypes of each gastrointestinal cancer are lacking, and consensus subtypes may be identified and confirmed in future cancer expression data. Second, reliable biomarkers corresponding to molecular subtypes to predict the response to current therapies are also lacking. Newer more effective approaches should be developed and applied in the detailed characterization of intra- and intertumoral heterogeneity, such as scRNA-seq and relevant preclinical models. Further precise targeting of tumor-initiating steps and driving events according to subtype-specific biomarkers might serve as a novel therapeutic strategy in gastrointestinal cancer treatment. Finally, systematic tumor and liquid biopsy techniques should be developed to define signature molecules allowing delineation of the complete molecular profile and patient classification.

In summary, we provide an overview of molecular classifications from the CSC perspective that may facilitate improvement in the clinical management of patients with gastrointestinal malignancies and thus result in more favorable outcomes.

## Data Availability

Not applicable.

## Abbreviations

ADEX: Aberrantly differentiated endocrine-exocrine
CCS: Colon cancer subtype
CHD1L: Chromodomain-helicase-DNA-bindingprotein 1-like gene
CIMP: CpG island methylator phenotype
CLDN6-DM1: Anti-CLDN6 monoclonal antibody conjugated with cytotoxic agent (Mertansine) DM1
CMS: Consensus molecular subtype
CRC: Colorectal carcinoma
CSC: Cancer stem cell
EAC: Oesophageal adenocarcinomas
EMT: Epithelial-to-mesenchymal transition
EN: Endoderm
EP: Epithelial phenotype
EpCAM: Epithelial cell adhesion molecule
ESC: Embryonic stem cell
ESCC: Oesophageal squamous cell carcinomas
FU: 5-Fluorouracil
HB: Hepatoblasts
HC: Hepatocytes
HCC: Hepatocellular carcinoma
LP: Liver progenitor cell
MP: Mesenchymal phenotype
MSI: Microsatellite-instable
MSS: Microsattelite-stable
OS: Overal survival
PDAC: Pancreatic cancer
PDXs: Patient-derived xenografts
PH: Premature hepatocytes
PP: Pancreatic progenitor
QM-PDA: Quasi-mesenchymal-pancreatic ductal adenocarcinoma
